# Healthy and Unhealthy Dietary Patterns of Depressive Symptoms in Middle-Aged Women

**DOI:** 10.3390/nu16060776

**Published:** 2024-03-08

**Authors:** Ji-Young Choi, Seon-Joo Park, Hae-Jeung Lee

**Affiliations:** 1 Institute for Aging and Clinical Nutrition Research, Gachon University, Seongnam-si 13120, Republic of Korea; mayyou0514@naver.com; 2 Department of Food and Nutrition, College of Bionanotechnology, Gachon University, Seongnam-si 13120, Republic of Korea; 3 Department of Health Sciences and Technology, Gachon Advanced Institute for Health Science and Technology (GAIHST), Gachon University, Incheon 21999, Republic of Korea

**Keywords:** depressive symptoms, dietary patterns, middle-aged women

## Abstract

Depressive symptoms are a common menopausal feature in middle-aged women and are associated with dietary factors. This study aimed to determine the association between dietary patterns and depressive symptoms in 2190 Korean women aged 45–69 years. Depressive symptoms were screened using the Beck Depression Inventory-II (BDI-II), and food intake was examined using a food frequency questionnaire. Dietary patterns were derived from principal components analysis and identified two dietary patterns: a “healthy” dietary pattern (high intake of whole-grain rice, legumes, vegetables, fruits, and fish) and an “unhealthy” dietary pattern (high intake of noodles, dumplings, sweets, red meat, soda, and coffee). After adjusting for all confounding factors, those with the highest healthy dietary pattern scores had a 0.56-fold lower risk of depressive symptoms than those with the lowest score (Odds Ratio (OR) = 0.56, 95% confidence interval (CI): 0.37–0.84, *p* for trend = 0.006). Conversely, those with the highest unhealthy pattern scores had a 1.85-fold higher risk of depressive symptoms than that of those in the lowest quartile (OR = 1.85, 95% CI: 1.30–2.63, *p* for trend = 0.002). In middle-aged women, a dietary pattern of high intake of fiber-rich whole-grain rice, fruits, vegetables, and legumes may help prevent and manage depressive symptoms.

## 1. Introduction

Middle-aged women experience emotional changes, such as depression, anxiety, and sleep problems, due to postmenopausal hormonal changes [1,2]. Among these symptoms, depression is one of the most common in midlife women [3]. The Korea Health Insurance Review and Assessment Service reported that 40–59-year-old middle-aged women had a depression rate of 18.6% in 2021, which is approximately twice as high as the 9.5% prevalence among middle-aged men [4]. Many factors contribute to the high incidence of depression in middle-aged women, including physical symptoms of menopause, psychological and social changes, stress, negative thinking, changes in quality of life, and hormonal changes [5,6,7]. In particular, women in the menopausal transition period have been shown to have twice the risk of developing depressive symptoms than those who are not in this transitional phase [8].

The mechanisms underlying depression are not fully understood. It has been suggested that dysfunction of norepinephrine and serotonin systems [9], abnormal gut microbiome composition [10], and inflammatory cytokines [11] may be potential causes. Dietary factors have been reported as a major influence on the gut microbiome, serotonin neurotransmission, and anti-inflammatory activity [12,13,14,15]. A diet rich in nutrients and fibers can improve gut health and increase gut microbial diversity [16]. A diverse gut microbiome, which is involved in the Gut–Brain axis and tryptophan–serotonin metabolism, has been reported to regulate depression and anxiety [12].

A cross-sectional study of adult men and women in the United States found that those with the highest intake of healthy dietary patterns had a lower risk of depression than those with the lowest intake [17]. Another cross-sectional study conducted in South Korea revealed an inverse association between healthy dietary patterns and the risk of depression among 40–69-year-old middle-aged adults [18]. According to a case-control study of adult women in Iran, a healthy dietary pattern characterized by a high intake of vegetables, fruits, eggs, fish, and whole grains may reduce the risk of depression [19]. In a cohort study involving middle-aged women in Australia, adherence to the Mediterranean dietary pattern was linked to a decreased risk of depressive symptoms [20]. A review reported that a Mediterranean diet with a balanced intake of fruits, nuts, legumes, olive oil, and fish is associated with a lower risk of depression [21].

In contrast, a cross-sectional study in China reported that the highest intake of the western dietary pattern, characterized by high consumption of red and processed meats, snacks, and desserts, was associated with an increased risk of depressive symptoms compared with the lowest intake [22]. A study of middle-aged adults in Taiwan found that a dietary pattern characterized by a low intake of fish, legumes, vegetables, and fruits and a high intake of red meat, significantly increased the risk of depressive symptoms [23]. unhealthy dietary patterns, such as a high intake of red meat, ice cream, cakes, and desserts, were associated with higher levels of depressive symptoms in Australian middle-aged women [24].

Although several studies have analyzed the association between depression and dietary patterns, research on dietary patterns among middle-aged Korean women is lacking. Therefore, this study aimed to identify dietary patterns that influence depressive symptoms in middle-aged Korean women.

## 2. Materials and Methods

### 2.1. Participants

The participants of this study were 2201 middle-aged women between 45 and 69 years of age residing in Seoul and Gyeonggi-do, Republic of Korea. The participants were recruited through posters or banners in hospitals and health centers and all participants provided informed consent before participating in the study. Eleven participants with a daily energy intake <500 kcal or >3500 kcal were excluded from the analysis [25], and the final analysis was conducted on 2190 participants. This study was approved by the Institutional Review Board of the Gachon University Gil Medical Center (GDIRB2016-271).

### 2.2. Methods

#### 2.2.1. Screening for Depressive Symptoms

Depressive symptoms were classified using the Beck Depression Inventory-II (BDI-II). The BDI-II consists of 21 items based on the DSM-IV (Diagnostic and Statistical Manual of Mental Disorders, 4th edition) diagnostic criteria for depressive disorders, with scores of 14–19 categorized as mild depression, 20–28 as moderate depression, and 29–63 as severe depression [26]. In this study, depressive symptoms were defined as a BDI-II score of 14 or higher.

#### 2.2.2. Covariates

General characteristics such as age, marital status, household type, income level, education, and occupation type were collected.

Marital status was classified as ‘married’ or ‘other’, and household type was classified as ‘living alone‘, ‘living with family’, or ‘other’. Income levels were classified as ‘less than 1,000,000 Won’, ‘more than 1,000,000 Won but less than 2,000,000 Won’, ‘more than 2,000,000 Won but less than 4,000,000 Won’, or ‘more than 4,000,000 Won’. Education level was classified as ‘less than elementary school’, ‘middle school’, ‘high school’, or ‘high school or higher’ and occupation was classified as ‘white-collar worker’, ‘service worker’, ‘blue-collar worker’, or ‘other’.

The body mass index (BMI) was calculated by dividing the participants’ weight (kg) by the square of their height (meter). Daily sleep time was categorized as ‘less than 6 hours’, ‘more than 6 h but less than 8 hours’, or ‘more than 8 hours’, following the guidelines of the Korean Society of Sleep Medicine [27]. Drinking status was categorized as ’non-drinking’, ‘less than once a week’, ‘2–3 times a week’, or ‘more than 4 times a week’. Smoking status was categorized as ‘non-smoking’, ‘former smoking’, or ‘current smoking’. Physical activities were classified as ‘low active‘, ‘active’, or ‘highly active’. Stress was classified as ‘not feeling at all’, ‘feeling a little’, ‘feeling a lot’, or ‘feeling very much’. Participants were categorized as having a history of disease if they were diagnosed by a physician with at least one of the following conditions: hypertension, diabetes, hyperlipidemia, heart disease, arthritis, or cancer. Participants were categorized as having a family history of depression if at least one of their father, mother, brother, or sister was diagnosed with depression by a physician. Participants who had not menstruated for the past 12 months were classified as ‘menopausal’.

#### 2.2.3. Dietary Assessment

The frequency of food intake was measured using a questionnaire with 108 food items based on a previously developed SQ-FFQ with some modifications [28]. The frequency of intake of the 108 foods included in the FFQ was classified into the following nine categories: ‘rarely’, ‘once a month’, ‘2–3 times a month’, ‘once a week’, ‘2–4 times a week’, ‘5–6 times a week’, ‘once a day’, ‘twice a day’, and ‘3 times a day’. Food intake was examined at 0.5, 1, and 1.5 times the baseline amount. The daily intake of energy, protein, fat, carbohydrates, fiber, calcium, phosphorus, iron, sodium, vitamin K, vitamin A, carotene, retinol, vitamin B_1_, vitamin B_2_, niacin, vitamin C, saturated fatty acids, monounsaturated fatty acids, and polyunsaturated fatty acids were calculated using a food composition database developed by the Rural Development Administration [29].

#### 2.2.4. Statistical Analysis

For factor analysis, the 108 food items were classified into 27 food groups based on similarities in their nutritional content and characteristics (Table 1). A factor analysis was performed using the weekly frequency of 108 food items intake. The principal component method and varimax rotation were used for factor extraction. To determine the number of factors to retain, we assessed the eigenvalues (>2), scree test plots, and interpretability of the factors. Each dietary pattern’s factor score was calculated by summing food consumption weighted by factor loading, and the factor scores were categorized into quartiles [17].

Among the general characteristics of the study participants, continuous variables were presented as means and standard errors using independent sample *t*-tests, whereas categorical variables were presented as frequencies and percentages using Chi-square tests.

Generalized linear models adjusted for energy intake were used to compare the weekly frequency of consumption of food groups and the daily intake of nutrients according to the quartiles of dietary pattern factor scores, with Scheffe’s post hoc tests for significance.

To analyze the association between dietary patterns and depressive symptoms, participants were divided into quartiles based on dietary pattern factor scores, and multiple logistic regression analyses were performed to obtain odds ratio (OR) and 95% confidence interval (CI). Model 1 was adjusted for age. Model 2 was adjusted for BMI, family history of depression, and chronic diseases to Model 1. Model 3 added socioeconomic factors such as household type, income level, marriage, education level, and occupation to Model 2. Model 4 added health behaviors such as drinking, smoking, sleep time, stress level, physical activity, and daily energy intake to Model 3. Confounding variables were defined as those with a *p*-value < 0.1 through stepwise and simple logistic regression analysis. To assess multicollinearity among the confounding variables, the Variance Inflation Factor (VIF) was examined, and it was found that all confounding variables had a VIF of 10 or less.

All statistical analyses were performed using the SAS (Statistical Analysis System version 9.4; SAS Institute, Cary, NC, USA) program, and statistical significance was set at *p* < 0.05.

## 3. Results

Among the 2190 participants, 478 (22.2%) with a BDI-II score ≥14 were classified into the depressive symptom group. The differences in the general characteristics between the non-depressive symptom and depressive symptom groups are shown in Table 2. In terms of the BDI-II score, the non-depressive symptom and depressive symptom groups had an average of 5.6 ± 0.1 and 21.0 ± 0.3 points, respectively(*p* < 0.0001). Compared with the non-depressive symptom group, the depressive symptom group had a higher percentage of individuals who were single and living alone, lower incomes, less sleep, a higher frequency of weekly alcohol consumption, and a significantly higher percentage of current smokers. They were also less physically active and had significantly higher stress levels, a higher prevalence of chronic diseases, and a family history of depression.

Two dietary patterns were extracted using factor analysis with factor loadings for each food group. The factor loading is the correlation coefficient between a principal component and a food group, where a higher factor loading value indicates a stronger correlation between the food group and the principal component [30]. A positive factor loading value means that the food group is positively correlated with the dietary pattern, while a negative factor loading value means that the food group is negatively correlated with the dietary pattern [31]. A “healthy” dietary pattern was characterized by a high intake of whole-grain rice, soybean products, nuts, vegetables, fruits, mushrooms, fish, seaweed, milk, and dairy products. An “unhealthy” dietary pattern was characterized by a high intake of refined grains, noodles, snacks, and meat and a low intake of whole-grain rice. The factor loading values for each food group according to dietary patterns are presented in Table 3.

The results of daily nutrient intake and food frequency according to quartiles of the “healthy” dietary pattern factor score are present in Table 4. The highest quartile group (Q4) of the healthy dietary pattern had a significantly higher daily intake of all nutrients, except carbohydrates, than the lowest quartile group (Q1) (*p* < 0.001). They were also significantly more likely to consume beans, mixed grains, and foods made from beans, nuts, vegetables, fruits, mushrooms, eggs, fish, seaweed, and dairy products than those in the lowest quartile of the healthy dietary pattern. Conversely, those with a healthier dietary pattern were less likely to consume white rice, refined cereals, noodles, dumplings, bread, cakes, snacks, chocolate, soda, coffee, or alcohol (*p* < 0.05).

Table 5 presents a comparison of daily nutrient intake and food frequency according to quartiles of the “unhealthy” dietary pattern factor score. The highest quartile group (Q4) of unhealthy dietary pattern factor scores had higher energy and fat intake than the lowest quartile group (Q1). Additionally, those in the highest quartile of the unhealthy dietary pattern factor scores (Q4) were significantly more inclined to consume white rice, refined grains, noodles, dumplings, bread, cakes, snacks, chocolate, meat, soda, coffee, and alcohol. However, they were significantly less inclined to consume beans, mixed grains, nuts, mushrooms, fruits, eggs, milk, and dairy products (*p* < 0.05).

The associations between the dietary pattern factor scores and depressive symptoms are shown in Table 6. In model 4, adjusted for age, BMI, family history of depression, chronic disease, household type, household income, marital status, education, occupation, alcohol consumption, smoking, sleep duration, stress, physical activity, and energy intake, the OR for the highest quartile of the “healthy” dietary pattern factor score showed a 0.56-fold lower risk of depressive symptoms compared to the lowest quartile (OR = 0.56, 95% CI: 0.37–0.84, *p* for trend=0.006). Conversely, after adjusting for all confounding factors, the OR for the highest quartile of the “unhealthy” dietary pattern factor score showed a 1.85-fold increased risk of depressive symptoms compared to the lowest quartile (OR = 1.85, 95% CI: 1.30–2.63, *p* for trend = 0.002).

## 4. Discussion

This study aimed to investigate the association between dietary patterns and depressive symptoms. After adjusting all confounders, a “healthy” dietary pattern, characterized by a high intake of multigrain rice, legumes, nuts, vegetables and fruits, fish, seaweed, milk, and dairy products, was significantly associated with a reduced risk of depressive symptoms. In contrast, an “unhealthy” dietary pattern, such as high consumption of bread, cakes, snacks, chocolate, soda, coffee, and alcohol, has been shown to increase the risk of depressive symptoms.

A study analyzing the association between depression and dietary patterns in middle-aged Korean adults found that those with the highest healthy dietary pattern scores (higher intake of vegetables, legumes, mushrooms, seaweed, white fish, and fruits and a lower intake of white rice) had a 0.59 times lower risk of depression than those with the lowest scores. In contrast, those with the highest unhealthy dietary pattern scores, which included a high intake of white rice, red meat, ramen, noodles, bread, and coffee, had a 1.65 times higher risk of depression than those with the lowest scores [18]. In the GAZEL cohort study, 12,000 adults aged 35–50 years were followed up for approximately 10 years. Among women, those with the highest traditional dietary pattern score and a high intake of fish and fruit had a 0.63-fold lower risk of depressive symptoms than those with the lowest dietary pattern scores. Similarly, women with the highest healthy eating score, characterized by high vegetable intake, had a 0.75 times lower risk of depressive symptoms than those with the lowest score. [32]. The SUN Cohort Study, which followed nearly 10,000 participants for 4 years, found that individuals with the highest adherence to the Mediterranean diet had a 0.58 times lower risk of depression than those with the lowest adherence. A higher intake of fruits, nuts, and legumes is also associated with a reduced risk of depression, whereas a high intake of high-fat dairy and red meat is associated with a higher risk of depression [33].

A meta-analysis reviewing 16 studies of dietary interventions, such as limiting the intake of fat, red meat, refined sugar, and excess calories, or adopting a Mediterranean diet, found that adherence to a healthy diet was associated with a significant reduction in depression symptoms (Hedges’ g = 0.275, 95% CI: 0.10–0.45) [34]. Another meta-study of 21 studies from 10 countries found that individuals adhering to a healthy dietary pattern, which is characterized by the high consumption of vegetables, fruits, whole grains, olive oil, and beans, had a lower risk of depression than those who did not (OR = 0.64, 95% CI: 0.57–0.72, *p* < 0.0001). Conversely, individuals with Western dietary patterns, characterized by a high intake of processed meats, refined grains, sugars, and butter, were found to have a higher risk of depression (OR = 1.18, CI: 1.05–1.34, *p* = 0.006) [35].

However, the mechanisms underlying depression are not yet clearly understood. The monoamine hypothesis, one of the hypotheses of depression etiology, suggests that prolonged exposure to inflammatory cytokines can lead to the dysfunction of monoamine neurotransmitters such as norepinephrine and dopamine [36,37]. This inflammatory response may affect the composition of the gut microbiome, potentially contributing to the development of depression [38]. A healthy gut microbiome positively affects mental health, including depression and anxiety, by influencing neurotransmitter synthesis, the myelination of neurons in the prefrontal cortex, and hippocampal development [12,16,39,40]. Eating a healthy diet is a major factor that positively influences the gut microbiome [41]. Dietary fiber, a key component of a healthy diet, is broken down into short-chain fatty acids by the gut microbiota, which not only regulates inflammation but also reduces depression through the production of γ-aminobutyric acid and serotonin [42].

Omega-3 fatty acids, phospholipids, cholesterol, niacin, folate, vitamin B_6_, vitamin B_12_, and antioxidant vitamins are known to have a positive impact on mental health [43]. Melatonin, tryptophan, taurine, and omega-3 fatty acids have been reported to promote a beneficial microbial environment in the gut [44]. Tryptophan is an essential amino acid required for the synthesis of serotonin, melatonin, and niacin and is found in foods such as milk and dairy products, fish, eggs, bananas, tofu, and nuts. Low tryptophan intake may impair serotonin synthesis in the brain, causing anxiety and depression [45]. Western dietary patterns, which are high in refined grains, sugars, and saturated fatty acids, reduce the diversity of intestinal microorganisms [46]. In particular, excessive consumption of added sugars can disrupt the gut microbial environment, leading to inflammatory responses, insulin resistance, oxidative stress, endorphin and dopamine dysregulation, and ultimately, depression [47,48]. In our study, participants with healthy eating habits who ate more fiber-rich foods tended to be less depressed, likely due to the beneficial effects of these gut flora.

This study has several limitations. First, depressive symptoms were assessed using a self-reporting questionnaire, which may lead to underreporting of mental health status or inaccurate perceptions of one’s condition, thereby limiting the accuracy of determining depression. Second, due to the cross-sectional study design, a causal relationship between depressive symptoms and dietary patterns could not be clearly determined. Nevertheless, this study is significant because it analyzed the association between depressive symptoms and dietary patterns among middle-aged Korean women. In addition, the participants’ food intake was calculated using a validated food frequency survey that allowed for the recording of food intake over time. Further research with larger sample sizes, objective measures of depressive symptoms, and long-term follow-ups is required to confirm the association between depressive symptoms and dietary patterns.

## 5. Conclusions

Healthy dietary patterns that involve consuming a variety of foods such as whole grains, legumes, nuts, vegetables and fruits, fish, seafood, and milk and dairy products reduce the risk of depressive symptoms. These foods are high in fiber, which appears to reduce depressive symptoms by regulating neurotransmitters and creating a beneficial microbial environment. In contrast, unhealthy dietary patterns involving the consumption of foods such as cakes, cookies, chocolate, carbonated drinks, coffee, and meat increased the risk of depressive symptoms in Korean middle-aged women. Therefore, a diet that includes a variety of fiber-rich foods is important for preventing and managing depressive symptoms in middle-aged women. To prevent depression, it is important to educate the public to increase their intake of fruits, vegetables, and whole grains, and to promote policies to increase the consumption of these foods.

## Figures and Tables

**Table 1:** Food grouping used in dietary pattern analysis.

Food Groups	Foods Included in the Food Group
Rice, rice flour, porridge, scorched rice, rice cakes	Well-milled cooked rice, Porridge, Mixed grain powder, Rice cakes
Whole-grain rice	Cooked rice with soybean, Cooked rice with black rice, Cooked rice with other cereals
Noodles and dumplings	Noodle, Udon, Kalguksu, Jajangmyeon, Jambbong, Cold noodles, Buckwheat noodles, Dumpling, Dumpling with soup
Bread and Cakes	White bread, Bread with red bean, Cream bread, Castella, Cake
Snacks, candies, and chocolate	Candy, Chocolate, Snacks, Cookies
Potato and sweet potato	Potato, Sweet potato, Corn, Starch jelly (acorn jelly, mung bean jelly), Starch vermicelli (Japchae)
Soybean, tofu, bean paste, and soy milk	Soybean milk, Tofu and tofu dishes, Soybean paste and soup/stew with soybean paste, Legumes
Peanut, almond, and pine nut	Peanuts, Walnuts, Pine nuts, Almonds
Kimchi	Cabbage kimchi, Kimchi stew, Stir-fried kimchi, Radish kimchi, Green onion kimchi, Leek kimchi, Leaf mustard kimchi, Other kimchi
Green and yellow vegetables	Lettuce, Sesame, Green pepper, Carrots, Spinach, Dried radish leaves, Green pumpkin, Cucumber, Pumpkin
Light-colored vegetables	Garlic, Onions, Cabbage, Bean sprout, Deoduck and Doraji (kinds of white root)
Pickles, salted fish	Korean-style pickles, salt-fermented fish
Mushrooms	Mushroom and mushroom dishes
Fruits	Apple, Tomato, Cherry tomato, Banana, Kiwi, Strawberry, Melon, Korean melon, Watermelon, Peach, Grape, Pear/pear juice, Persimmon/hard persimmon/dried persimmon, Tangerine, Orange, Orange juice, Tomato juice, grape juice
Meat	Pork and pork dishes (grilled pork belly, stir-fried pork, stew, ham/sausage), Beef and beef dishes (beef-bone soup, bulgogi, rib), Edible viscera, Fried chicken and chicken stew
Egg	Fried egg, Steamed egg, Boiled egg
White fish	Alaska pollack, Yellow croaker, Sea bream, Flat fish
Blue-backed fish	Mackerel, Pacific saury, Spanish mackerel, Canned tuna
Anchovy	Dried anchovy, Stir-fried anchovies, Dried sliced whitefish
Squid and small octopus	Seafood (crab, squid, small octopus, shellfish, shrimp) and seafood stew
Fish cake and imitation crab meat	Fish cake, Imitation crab meat
Laver, brown kelp, and sea mustard	Sea mustard and sea mustard dishes, Kelp, Dried green laver
Milk, yogurt, ice cream, and cheese	Milk, Yogurt, Cheese
Soda	Carbonated drinks
Coffee	Black coffee, Coffee premix
Tea	Green tea, Black tea, Citron tea, Other teas
Alcoholic drink	Soju, Beer, Rice wine (Makgeolli), Fruit wine

**Table 2:** General characteristics of subjects according to the depressive symptom status.

	Non-Depressive Symptoms (*n* = 1703)		Depressive Symptoms (*n* = 487)		*p*-Value ^1^
Mean (SE)					
Age (years)	58.2	(0.1)	57.8	(0.3)	0.1669
Height	156.2	(0.1)	156.7	(0.2)	0.0527
Weight	59.0	(0.2)	58.9	(0.4)	0.7452
BMI	24.2	(0.1)	24.0	(0.2)	0.2894
BDI-II Score	5.6	(0.1)	21.0	(0.3)	<0.0001
Intake of energy	1352.2	(9.1)	1315.8	(16.9)	0.0586
N (%)					
Marital status					0.0049
Married	1401	(82.3)	373	(76.6)	
Other	302	(17.7)	114	(23.4)	
Type of household					0.0357
Alone	160	(9.4)	61	(12.5)	
With family	1507	(88.5)	410	(84.2)	
Other	36	(2.1)	16	(3.3)	
Household income					<0.0001
<1,000,000	123	(7.2)	61	(12.5)	
1,000,000 ≤~< 2,000,000	324	(19.0)	126	(25.9)	
2,000,000 ≤~< 4,000,000	635	(37.3)	166	(34.1)	
≥4,000,000	621	(36.5)	134	(27.5)	
Education					0.1795
Elementary school	237	(13.9)	85	(17.5)	
Middle school	448	(26.3)	121	(24.9)	
High school	751	(44.1)	216	(44.4)	
College and higher	267	(15.7)	65	(13.4)	
Job					0.3293
White collar	141	(8.3)	28	(5.8)	
Service worker	383	(22.5)	114	(23.4)	
Blue collar	158	(9.3)	47	(9.7)	
Other	1021	(60.0)	298	(61.2)	
Sleep duration					0.0414
<6 h/day	281	(16.5)	104	(21.4)	
6–8 h/day	1122	(65.9)	298	(61.2)	
≥8 h/day	300	(17.6)	85	(17.5)	
Alcohol frequency (last 1 year)					0.0148
No	1210	(71.1)	347	(71.3)	
≤1/week	376	(22.1)	93	(19.1)	
2~3/week	106	(6.2)	37	(7.6)	
≥4/week	11	(0.7)	10	(2.1)	
Smoking status					
Non smoker	1661	(97.5)	458	(94.1)	
Ex-smoker	12	(0.7)	6	(1.2)	
Current smoker	30	(1.8)	23	(4.7)	
Physical activity					<0.0001
Low	223	(13.1)	121	(24.9)	
Median	1059	(62.2)	283	(58.1)	
High	421	(24.7)	83	(17.0)	
Stress					<0.0001
Rarely	514	(30.2)	35	(7.2)	
A little	909	(53.4)	179	(36.8)	
A lot	269	(15.8)	251	(51.5)	
Very much	11	(0.7)	22	(4.5)	
Chronic disease ^2^					0.0227
No	956	(56.1)	245	(50.3)	
Yes	747	(43.9)	242	(49.7)	
Family history of depression					0.0012
No	1681	(98.7)	470	(96.5)	
Yes	22	(1.3)	17	(3.5)	
Menopausal status					0.8091
No	192	(11.3)	53	(10.9)	
Yes	1511	(88.7)	434	(89.1)	

^1^*p*-value: chi-square test or Student’s *t*-test. ^2^ Chronic diseases include hypertension, diabetes, hyperlipidemia, cardiovascular disease, arthritis, and cancers.

**Table 3:** Factor loading matrix of the two dietary patterns.

Food Group ^1^	Healthy	Unhealthy
Rice, rice flour, porridge, scorched rice, rice cakes	−0.21 ^2^	0.68
Whole-grain rice	0.27	−0.68
noodles and dumplings		0.52
Bread and Cakes		0.31
Snacks, candies, and chocolate		0.40
Potato and sweet potato	0.40	
Soybean, tofu, bean paste, and soy milk	0.67	
Peanut, almond, and pine nut	0.43	
Kimchi	0.34	
Green and yellow vegetables	0.71	
Light colored vegetables	0.62	
Pickles, salted fish		
Mushrooms	0.48	
Fruits	0.58	
Meat	0.37	0.43
Egg	0.38	
White fish	0.53	0.20
Blue-backed fish	0.53	
Anchovy	0.40	
Squid and small octopus	0.38	0.31
Fish cake and imitation crab meat	0.28	0.37
Laver, brown kelp, and sea mustard	0.46	
Milk, yogurt, ice cream, and cheese	0.44	
Soda		0.38
Coffee		0.38
Tea	0.20	
Alcoholic drink		0.21

^1^ Food groups with absolute values < 0.20 are not shown for simplicity. ^2^ Correlation coefficients (r). Factor loadings represent the magnitude and direction of association with factors (dietary patterns) and can range from −1.0 to 1.0.

**Table 4:** Dietary intake of participants according to the healthy dietary patterns score quartiles.

	Q1 (Lowest) (*n* = 547)		Q2 (*n* = 548)		Q3 (*n* = 548)		Q4 (Highest) (*n* = 547)		*p*-Trend
Nutrient intake ^1,2^									
Energy (kcal/d)	1066.7	± 12.7 ^d^	1220.4	± 12.7 ^c^	1406.6	± 12.7 ^b^	1682.9	± 12.7 ^a^	<0.0001
Protein (g/d)	39.9	± 0.3 ^d^	43.2	± 0.2 ^c^	45.7	± 0.2 ^b^	53.2	± 0.3 ^a^	<0.0001
Fat (g/d)	24.1	± 0.3 ^d^	27.5	± 0.3 ^c^	29.8	± 0.3 ^b^	36.6	± 0.4 ^a^	<0.0001
Carbohydrate (g/d)	230.8	± 1.0 ^a^	223.8	± 0.9 ^b^	218.7	± 0.9 ^c^	199.7	± 1.0 ^d^	<0.0001
Fiber (g/d)	4.3	± 0.1 ^d^	4.9	± 0.1 ^c^	5.6	± 0.1 ^b^	6.6	± 0.1 ^a^	<0.0001
Calcium (mg/d)	297.0	± 5.3 ^d^	366.9	± 4.9 ^c^	409.4	± 4.8 ^b^	513.8	± 5.5 ^a^	<0.0001
Phosphorous (mg/d)	645.2	± 5.0 ^d^	727.0	± 4.6 ^c^	783.0	± 4.5 ^b^	928.9	± 5.2 ^a^	<0.0001
Fe (mg/d)	9.0	± 0.1 ^d^	9.9	± 0.1 ^c^	10.9	± 0.1 ^b^	13.0	± 0.1 ^a^	<0.0001
Sodium (mg/d)	2255.3	± 38.4	2613.8	± 35.5 ^c^	2957.9	± 34.9 ^b^	3756.2	± 40.0 ^a^	<0.0001
K (mg/d)	1646.5	± 15.2 ^d^	1910.5	± 14.1 ^c^	2155.1	± 13.8 ^b^	2641.2	± 15.9 ^a^	<0.0001
Vitamin A (RE/d)	349.4	± 7.8 ^d^	431.3	± 7.2 ^c^	514.4	± 7.1 ^b^	721.8	± 8.2 ^a^	<0.0001
Carotene (μg/d)	1643.0	± 44.9 ^d^	2041.4	± 41.5 ^c^	2498.2	± 40.8 ^b^	3587.2	± 46.8 ^a^	<0.0001
Retinol (μg/d)	65.7	± 2.5 ^d^	78.9	± 2.3 ^c^	84.6	± 2.3 ^b^	106.4	± 2.6 ^a^	<0.0001
Vitamin B_1_ (mg/d)	1.2	± 0.0 ^d^	1.2	± 0.0 ^c^	1.3	± 0.0 ^b^	1.5	± 0.0 ^a^	<0.0001
Vitamin B_2_ (mg/d)	0.7	± 0.0 ^d^	0.8	± 0.0 ^c^	0.9	± 0.0 ^b^	1.2	± 0.0 ^a^	<0.0001
Niacin (mg/d)	8.4	± 0.1 ^d^	9.0	± 0.1 ^c^	9.8	± 0.1 ^b^	11.5	± 0.1 ^a^	<0.0001
Vitamin C (mg/d)	40.1	± 1.3 ^d^	53.5	± 1.2 ^c^	68.2	± 1.2 ^b^	89.2	± 1.4 ^a^	<0.0001
saturated fatty acid (mg/d)	7.3	± 0.1 ^d^	8.1	± 0.1 ^c^	8.4	± 0.1 ^b^	9.8	± 0.1 ^a^	<0.0001
monounsaturated fatty acid (mg/d)	7.7	± 0.1 ^d^	8.7	± 0.1 ^c^	9.5	± 0.1 ^b^	11.6	± 0.1 ^a^	<0.0001
polyunsaturated fatty acid (mg/d)	5.9	± 0.1 ^d^	7.0	± 0.1 ^c^	8.0	± 0.1 ^b^	10.5	± 0.1 ^a^	<0.0001
Food group intake ^1,2^ (times/week)									
Rice, rice flour, porridge, scorched rice, rice cakes	9.8	± 0.3 ^a^	5.8	± 0.3 ^b^	4.0	± 0.3 ^c^	1.4	± 0.3 ^d^	<0.0001
Boiled rice and cereals	10.3	± 0.4 ^d^	14.3	± 0.3 ^c^	15.7	± 0.3 ^b^	17.0	± 0.4 ^a^	<0.0001
noodles and dumplings	1.2	± 0.1 ^a^	1.1	± 0.0 ^ab^	1.0	± 0.0 ^bc^	0.8	± 0.1 ^c^	<0.0001
Bread and Cakes	1.0	± 0.1 ^a^	0.8	± 0.1 ^ab^	0.7	± 0.1 ^b^	0.4	± 0.1 ^c^	<0.0001
Snacks, candies, and chocolate	1.8	± 0.1 ^a^	1.5	± 0.1 ^ab^	1.3	± 0.1 ^bc^	0.8	± 0.1 ^c^	<0.0001
Potato and sweet potato	1.4	± 0.1 ^d^	1.7	± 0.1 ^c^	2.0	± 0.1 ^b^	2.7	± 0.1 ^a^	<0.0001
Soybean, tofu, bean paste, and soy milk	3.5	± 0.2 ^d^	4.9	± 0.2 ^c^	6.1	± 0.2 ^b^	9.7	± 0.2 ^a^	<0.0001
Peanut, almond, and pine nut	0.9	± 0.1 ^d^	2.0	± 0.1 ^c^	3.0	± 0.1 ^b^	4.2	± 0.1 ^a^	<0.0001
Kimchi	14.8	± 0.5 ^c^	17.6	± 0.4 ^b^	19.1	± 0.4 ^b^	21.1	± 0.5 ^a^	<0.0001
Green and yellow vegetables	4.2	± 0.2 ^d^	6.2	± 0.2 ^c^	7.8	± 0.2 ^b^	12.3	± 0.2 ^a^	<0.0001
Light colored vegetables	2.7	± 0.1 ^d^	4.1	± 0.1 ^c^	5.0	± 0.1 ^b^	7.8	± 0.2 ^a^	<0.0001
Pickles, salted fish	0.4	± 0.1 ^c^	0.6	± 0.1 ^bc^	0.9	± 0.1 ^ab^	1.2	± 0.1 ^a^	<0.0001
Mushrooms	0.6	± 0.1 ^d^	0.8	± 0.1 ^c^	1.3	± 0.1 ^b^	2.2	± 0.1 ^a^	<0.0001
Fruits	4.8	± 0.2 ^d^	6.7	± 0.2 ^c^	9.5	± 0.2 ^b^	13.2	± 0.3 ^a^	<0.0001
Meat	2.5	± 0.1 ^ab^	2.4	± 0.1 ^b^	2.4	± 0.1 ^b^	2.8	± 0.1 ^a^	0.0088
Egg	2.4	± 0.1 ^c^	2.9	± 0.1 ^c^	3.5	± 0.1 ^b^	4.5	± 0.2 ^a^	<0.0001
White fish	0.6	± 0.1 ^c^	0.8	± 0.1 ^c^	1.0	± 0.1 ^b^	1.9	± 0.1 ^a^	<0.0001
Blue-backed fish	0.4	± 0.0 ^d^	0.6	± 0.0 ^c^	0.8	± 0.0 ^b^	1.5	± 0.0 ^a^	<0.0001
Anchovy	0.9	± 0.1 ^d^	1.8	± 0.1 ^c^	2.7	± 0.1 ^b^	3.9	± 0.2 ^a^	<0.0001
Squid and small octopus	0.3	± 0.0 ^c^	0.4	± 0.0 ^c^	0.5	± 0.0 ^b^	0.7	± 0.0 ^a^	<0.0001
Fish cake and imitation crab meat	0.2	± 0.0 ^b^	0.3	± 0.0 ^b^	0.3	± 0.0 ^b^	0.5	± 0.0 ^a^	<0.0001
Laver, brown kelp, and sea mustard	1.8	± 0.2 ^d^	2.5	± 0.1 ^c^	3.6	± 0.1 ^b^	5.1	± 0.2 ^a^	<0.0001
Milk, yogurt, ice cream, and cheese	4.0	± 0.3 ^c^	5.8	± 0.2 ^b^	6.4	± 0.2 ^b^	8.4	± 0.3 ^a^	<0.0001
Soda	0.3	± 0.0 ^a^	0.2	± 0.0 ^ab^	0.1	± 0.0 ^b^	0.1	± 0.0 ^b^	0.0012
Coffee	10.0	± 0.3 ^a^	9.3	± 0.3 ^ab^	8.1	± 0.3 ^bc^	7.8	± 0.4 ^c^	<0.0001
Tea	0.5	± 0.1 ^c^	0.8	± 0.1 ^c^	1.6	± 0.1 ^b^	2.2	± 0.1 ^a^	<0.0001
Alcoholic drink	1.3	± 0.1 ^a^	0.7	± 0.1 ^b^	0.5	± 0.1 ^bc^	0.3	± 0.1 ^c^	<0.0001

^1^ Values are mean ± standard error and calculated using the generalized linear model. ^2^ Nutrient and food intake were adjusted for energy intake. ^a–d^: Values with different superscripts within a row are significantly different (*p* < 0.05) by Scheffe’s post hoc test.

**Table 5:** Dietary intake of participants according to the unhealthy dietary patterns score quartiles.

	Q1 (Lowest) (*n* = 547)		Q2 (*n* = 548)		Q3 (*n* = 548)		Q4 (Highest) (*n* = 547)		*p*-Trend
Nutrient intake ^1,2^									
Energy (kcal/d)	1259.6	± 15.7 ^cd^	1287.9	± 15.7 ^c^	1362.5	± 15.7 ^b^	1466.6	± 15.7 ^a^	<0.0001
Protein (g/d)	45.5	± 0.3 ^ab^	45.7	± 0.3 ^ab^	46.0	± 0.3 ^a^	44.8	± 0.3 ^b^	0.0234
Fat (g/d)	28.5	± 0.3 ^b^	28.8	± 0.3 ^b^	30.4	± 0.3 ^a^	30.4	± 0.3 ^a^	<0.0001
Carbohydrate (g/d)	222.9	± 1.0 ^a^	220.5	± 1.0 ^a^	214.8	± 1.0 ^b^	214.8	± 1.0 ^b^	<0.0001
Fiber (g/d)	5.6	± 0.1 ^a^	5.6	± 0.1 ^a^	5.3	± 0.1 ^b^	5.0	± 0.1 ^c^	<0.0001
Calcium (mg/d)	426.7	± 5.4 ^a^	399.2	± 5.4 ^b^	396.7	± 5.4 ^b^	364.6	± 5.5 ^c^	<0.0001
Phosphorous (mg/d)	808.4	± 5.6 ^a^	782.9	± 5.6 ^b^	774.9	± 5.5 ^b^	717.9	± 5.6 ^c^	<0.0001
Fe (mg/d)	10.9	± 0.1 ^ab^	11.0	± 0.1 ^a^	10.6	± 0.1 ^bc^	10.3	± 0.1 ^c^	<0.0001
Sodium (mg/d)	2780.8	± 39.7 ^b^	2900.3	± 39.5 ^ab^	2915.3	± 39.4 ^ab^	2986.3	± 40.0 ^a^	0.0037
K (mg/d)	2196.6	± 18.2 ^a^	2135.1	± 18.1 ^ab^	2081.1	± 18.1 ^b^	1940.1	± 18.4 ^c^	<0.0001
Vitamin A (RE/d)	522.2	± 8.6	506.1	± 8.5	499.9	± 8.5	488.7	± 8.6	0.0512
Carotene (μg/d)	2503.9	± 47.9	2458.5	± 47.6	2407.8	± 47.5	2398.9	± 48.2	0.3853
Retinol (μg/d)	89.9	± 2.3 ^a^	83.3	± 2.3 ^ab^	85.4	± 2.3 ^ab^	77.1	± 2.3 ^b^	0.0016
Vitamin B_1_ (mg/d)	1.3	± 0.0 ^a^	1.3	± 0.0 ^a^	1.3	± 0.0 ^ab^	1.3	± 0.0 ^b^	0.0005
Vitamin B_2_ (mg/d)	0.9	± 0.0	0.9	± 0.0	0.9	± 0.0	0.9	± 0.0	0.1772
Niacin (mg/d)	9.5	± 0.1	9.7	± 0.1	9.7	± 0.1	9.7	± 0.1	0.4375
Vitamin C (mg/d)	71.0	± 1.3 ^a^	65.0	± 1.3 ^b^	62.2	± 1.3 ^b^	52.9	± 1.3 ^c^	<0.0001
saturated fatty acid (mg/d)	8.0	± 0.1 ^b^	8.1	± 0.1 ^b^	8.8	± 0.1 ^a^	8.8	± 0.1 ^a^	<0.0001
monounsaturated fatty acid (mg/d)	9.0	± 0.1 ^b^	9.1	± 0.1 ^b^	9.7	± 0.1 ^a^	9.7	± 0.1 ^a^	<0.0001
polyunsaturated fatty acid (mg/d)	8.0	± 0.1	7.8	± 0.1	7.9	± 0.1	7.7	± 0.1	0.1213
Food group intake ^1,2^ (times/week)									
Rice, rice flour, porridge, scorched rice, rice cakes	0.6	± 0.2 ^d^	1.7	± 0.2 ^c^	4.8	± 0.2 ^b^	13.9	± 0.2 ^a^	<0.0001
Boiled rice and cereals	20.4	± 0.2 ^a^	18.3	± 0.2 ^b^	13.7	± 0.2 ^c^	4.9	± 0.2 ^d^	<0.0001
noodles and dumplings	0.4	± 0.0 ^d^	0.9	± 0.0 ^c^	1.1	± 0.0 ^b^	1.6	± 0.0 ^a^	<0.0001
Bread and Cakes	0.3	± 0.1 ^c^	0.6	± 0.1 ^b^	0.8	± 0.1 ^b^	1.2	± 0.1 ^a^	<0.0001
Snacks, candies, and chocolate	0.5	± 0.1 ^c^	0.9	± 0.1 ^c^	1.4	± 0.1 ^b^	2.6	± 0.1 ^a^	<0.0001
Potato and sweet potato	1.7	± 0.1 ^b^	2.0	± 0.1 ^ab^	2.0	± 0.1 ^ab^	2.1	± 0.1 ^a^	0.0094
Soybean, tofu, bean paste, and soy milk	6.3	± 0.2	6.2	± 0.2	5.9	± 0.2	5.8	± 0.2	0.1591
Peanut, almond, and pine nut	3.8	± 0.1 ^a^	2.5	± 0.1 ^b^	2.3	± 0.1 ^b^	1.5	± 0.1 ^c^	<0.0001
Kimchi	19.9	± 0.4 ^a^	19.1	± 0.4 ^a^	17.2	± 0.4 ^b^	16.3	± 0.4 ^b^	<0.0001
Green and yellow vegetables	7.8	± 0.2	8.0	± 0.2	7.3	± 0.2	7.3	± 0.2	0.0452
Light colored vegetables	5.3	± 0.1	4.9	± 0.1	4.8	± 0.1	4.8	± 0.2	0.087
Pickles, salted fish	0.5	± 0.1 ^c^	0.7	± 0.1 ^bc^	0.9	± 0.1 ^ab^	1.1	± 0.1 ^a^	<0.0001
Mushrooms	1.5	± 0.1 ^a^	1.2	± 0.1 ^b^	1.2	± 0.1 ^b^	1.1	± 0.1 ^b^	0.0002
Fruits	10.3	± 0.2 ^a^	8.9	± 0.2 ^b^	8.4	± 0.2 ^b^	6.7	± 0.2 ^c^	<0.0001
Meat	1.7	± 0.1 ^d^	2.3	± 0.1 ^c^	2.8	± 0.1 ^b^	3.4	± 0.1 ^a^	<0.0001
Egg	3.7	± 0.1 ^a^	3.4	± 0.1 ^ab^	3.1	± 0.1 ^b^	3.0	± 0.1 ^b^	0.001
White fish	0.9	± 0.1 ^b^	1.0	± 0.1 ^ab^	1.2	± 0.1 ^a^	1.2	± 0.1 ^a^	<0.0001
Blue-backed fish	0.9	± 0.0	0.8	± 0.0	0.9	± 0.0	0.9	± 0.0	0.6289
Anchovy	3.2	± 0.1 ^a^	2.3	± 0.1 ^b^	2.1	± 0.1 ^b^	1.7	± 0.1 ^b^	<0.0001
Squid and small octopus	0.3	± 0.0 ^c^	0.4	± 0.0 ^b^	0.5	± 0.0 ^b^	0.6	± 0.0 ^a^	<0.0001
Fish cake and imitation crab meat	0.1	± 0.0 ^c^	0.2	± 0.0 ^b^	0.3	± 0.0 ^b^	0.6	± 0.0 ^a^	<0.0001
Laver, brown kelp, and sea mustard	3.4	± 0.1	3.2	± 0.1	3.2	± 0.1	3.2	± 0.1	0.8284
Milk, yogurt, ice cream, and cheese	7.2	± 0.2 ^a^	6.1	± 0.2 ^b^	6.4	± 0.2 ^ab^	4.8	± 0.2 ^c^	<0.0001
Soda	0.0	± 0.0 ^b^	0.1	± 0.0 ^b^	0.1	± 0.0 ^b^	0.4	± 0.0 ^a^	<0.0001
Coffee	4.7	± 0.3 ^d^	8.3	± 0.3 ^c^	10.1	± 0.3 ^b^	12.2	± 0.3 ^a^	<0.0001
Tea	1.5	± 0.1	1.2	± 0.1	1.2	± 0.1	1.2	± 0.1	0.4125
Alcoholic drink	0.2	± 0.1 ^b^	0.5	± 0.1 ^b^	1.0	± 0.1 ^a^	1.1	± 0.1 ^a^	<0.0001

^1^ Values are mean ± standard error and calculated using the generalized linear model. ^2^ Nutrient and food intake were adjusted for energy intake. ^a–d^: Values with different superscripts within a row are significantly different (*p* < 0.05) by Scheffe’s post-hoc test.

**Table 6:** Odds ratio and 95% confidence interval for depressive symptoms according to quartile of dietary pattern scores.

	All (*n* = 2190)	Case (*n* = 487)	Model 1 OR (95% CI)	Model 2 OR (95% CI)	Model 3 OR (95% CI)	Model 4 OR (95% CI)
Healthy dietary pattern						
Q1	547	166	1.00 (Reference)	1.00 (Reference)	1.00 (Reference)	1.00 (Reference)
Q2	548	121	0.65 (0.50–0.86)	0.64 (0.48–0.84)	0.67 (0.51–0.88)	0.73 (0.53–1.01)
Q3	548	106	0.56 (0.42–0.74)	0.55 (0.42–0.73)	0.59 (0.44–0.79)	0.64 (0.45–0.90)
Q4	547	94	0.48 (0.36–0.64)	0.48 (0.36–0.65)	0.54 (0.40–0.73)	0.56 (0.37–0.84)
	*p* for trend		<0.0001	<0.0001	<0.0001	0.006
Unhealthy dietary pattern						
Q1	547	90	1.00 (Reference)	1.00 (Reference)	1.00 (Reference)	1.00 (Reference)
Q2	548	123	1.47 (1.08–1.99)	1.48 (1.09–2.01)	1.50 (1.10–2.05)	1.43 (1.02–2.01)
Q3	548	117	1.38 (1.01–1.88)	1.41 (1.03–1.93)	1.47 (1.07–2.03)	1.55 (1.09–2.20)
Q4	547	157	2.04 (1.50–2.76)	2.08 (1.54–2.83)	2.14 (1.56–2.92)	1.85 (1.30–2.63)
	*p* for trend		<0.0001	<0.0001	<0.0001	0.002

Model 1 = Adjusted age. Model 2 = Adjusted model 1 + BMI, family history of depression, chronic disease, Model 3 = Adjusted model 2 + socioeconomic factors (type of household, household income, marriage status, education, job), Model 4 = Adjusted model 3 + health behavior (alcohol drinking, smoking, sleep-duration, stress, physical activity, energy [kcal]).

## Data Availability

Data are contained within the article.
